# Significant Electromechanical Characteristic Enhancement of Coaxial Electrospinning Core–Shell Fibers

**DOI:** 10.3390/polym14091739

**Published:** 2022-04-25

**Authors:** Duc-Nam Nguyen, Wonkyu Moon

**Affiliations:** 1 Department of Mechanical Engineering, Pohang University of Science and Technology (POSTECH), San 31, Hyojadong, Namgu, Pohang 37673, Kyungbuk, Korea; wkmoon@postech.ac.kr; 2 Faculty of Mechanical Engineering and Mechatronics, PHENIKAA University, Hanoi 12116, Vietnam; 3 PHENIKAA Research and Technology Institute (PRATI), A&A Green Phoenix Group JSC, No. 167 Hoang Ngan, Trung Hoa, Cau Giay, Hanoi 11313, Vietnam

**Keywords:** electrospinning, PBLG, PVDF, piezoelectric fibers, energy harvesting devices, implantable biosensors, electromechanical transducer, core–shell fibers

## Abstract

Electrospinning is a low-cost and straightforward method for producing various types of polymers in micro/nanofiber form. Among the various types of polymers, electrospun piezoelectric polymers have many potential applications. In this study, a new type of functional microfiber composed of poly(γ-benzyl-α,L-glutamate) (PBLG) and poly(vinylidene fluoride) (PVDF) with significantly enhanced electromechanical properties has been reported. Recently reported electrospun PBLG fibers exhibit polarity along the axial direction, while electrospun PVDF fibers have the highest net dipole moment in the transverse direction. Hence, a combination of PBLG and PVDF as a core–shell structure has been investigated in the present work. On polarization under a high voltage, enhancement in the net dipole moment in each material and the intramolecular conformation was observed. The piezoelectric coefficient of the electrospun PBLG/PVDF core–shell fibers was measured to be up to 68 pC N^−1^ (d_33_), and the voltage generation under longitudinal extension was 400 mVpp (peak-to-peak) at a frequency of 60 Hz, which is better than that of the electrospun homopolymer fibers. Such new types of functional materials can be used in various applications, such as sensors, actuators, smart materials, implantable biosensors, biomedical engineering devices, and energy harvesting devices.

## 1. Introduction

Over the past decades, the rapid advancements in piezoelectric polymer-based sensors, piezoelectric energy harvesters (PEHs), and piezoelectric energy nanogenerators (PENG) have promoted many applications ranging from wearable health monitoring devices and smart textiles to implantable medical electronics (IMEs) and bio-actuators [1,2,3,4,5,6,7,8,9]. Piezoelectric energy generators are currently a hot topic because of their special characteristics such as possible fabrication in small dimensions and compact structures, high reliability and sensitivity to small strains, and impressive high-density power output. Several piezoelectric materials such as ceramics, composites, and polymers have been widely studied and fabricated in the form of thin films, membranes, fiber mats, and layers to discover their potential. Functional nano/microfibers can be fabricated by various types of fabrication techniques, such as electrospinning (ES), force-spinning, mechano-electrospinning, and drawing [10,11,12]. T. Lei et al. [12] proved that poly(vinylidene fluoride) (PVDF) fibers produced by force-spinning are non-piezoelectric but have a high polar phase fraction, while PVDF fibers fabricated by electrospinning contain induced oriented dipoles normal to the thickness direction. Hence, ES is widely used for polymers that can be polarized in the presence of an electric field. This special condition promotes the orientation of the dipole moment or polar phase inside the structure. As a result, the amount of dipole moment or polar phases inside the material can greatly affect the piezoelectric coefficients and the electromechanical outcome.

**Figure 1:**
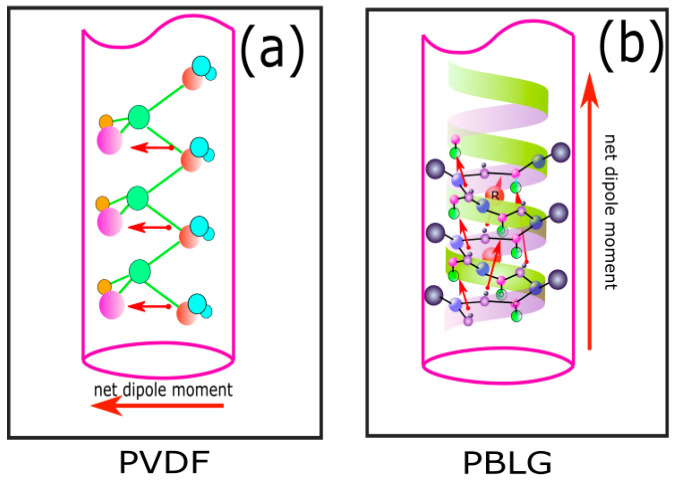
The orientation of the dipole moment (red arrows) in (**a**) an electrospun PVDF fiber and (**b**) an electrospun PBLG fiber. The net dipole moment reveals the largest polar direction in which the piezoelectric coefficient of a material is the largest.

Piezoelectric polymers have an average piezoelectric coefficient compared to other materials such as ceramic (PZT) and quartz. However, because of their light weight and high flexibility, ferroelectric polymers such as poly(vinylidene fluoride) (PVDF) have great potential in wearable electronic applications [7,13,14,15,16,17] Since the 1960s, poly(vinylidene fluoride) (PVDF) and its coherent polymers, such as poly(vinylidene fluoride–trifluoroethylene) (P(VD-TrFE)), have been widely applied in piezoelectric functional applications because of their high flexibility, low cost, and good electromechanical properties [18]. A number of studies have shown that PVDF and its coherent polymers contain semi-crystalline polymorphs, namely α, β, γ, and δ crystalline phases. Among these phases, the polar β-phase has the desired characteristics for electromechanical sensors and can be formed by several processing methods, such as mechanical stretching or electrical poling (Figure 1a) [19]. So far, an electrospun PVDF fiber-based composite has been reported to produce an output voltage up to 190 mVpp, a piezoelectric voltage constant up to g_31_ = 33.9 mV N^−1^, and g_33_ = 61.2 mV N^−1^ [20], while electrospun homopolymer P(VDF-TrFE) has exhibited a maximum sensitivity of 60.5 mV N^−1^ [21].

One of the newly discovered piezoelectric polymers is the polypeptide group, which contains a number of intermolecular dipole moments. Among the polypeptide polymers, poly(γ-benzyl-α,L-glutamate) (PBLG) and poly(γ-methyl-α,L-glutamate) (PMLG) have been extensively studied for many applications, such as biomedical engineering [22], organic memory [23], tissue and surface bioengineering, and biosensors [24]. In an α-helical polypeptide, the dipole moment is formed by parallel and directional alignments of hydrogen bonds along the helical axis, which are susceptible to the electric field (Figure 1b). The α-helical poly(α-amino acid) fibers, such as PBLG fibers fabricated by electrospinning (ES), have demonstrated notable electromechanical characteristics, such as a high piezoelectric charge constant d_33_ = 27 pC N^−1^ along the fiber axis, outstanding thermal stability, and stable dipole moment caused by the polarized α-helical structure. Farrar et al. [25] discovered the superior thermal stability of PBLG electrospun over 24 h at 100 °C. Nguyen et al. [26] verified the main contribution of parallel fibers to the overall piezoelectric coefficients by fabricating highly oriented electrospun PBLG fibers.

Electrospun fibers have been integrated into core–shell or composite structures to enhance the electromechanical characteristics of polymer microfibers. Lu et al. [5] fabricated electrospun core–shell fibers with P(VDF-TrFE) as the core material and a flexible conductive material as the shell. This type of core–shell fiber exhibited a high sensitivity of 60.81 mV N^−1^ and excellent durability for 15,000 cycles of compression. Sharma et al. [14] demonstrated that electrospun core–shell PEDOT:PSS/P(VDF-TrFE) exhibited a 4.5 times higher electrical gain intensity than that of electrospun homopolymer PVDF fibers. Core–shell nanofibers of PDMS/PVDF-HFP fabricated by Meng-Fang Lin et al. [27] generated a maximum power density of 0.9 W m^2^, which is sufficient to light up several hundred light-emitting diodes (LEDs) at the same time. On the other hand, the sensitivity of the electrospun PBLG integrated into the PBLG/PDMS composite was calculated as 615 mV N^−1^, which is far better than that of monolithic electrospun PBLG fibers (65 mV N^−1^) [28]. PBLG is classified as a liquid crystal polymer (LCP) that exhibits important functions among other highly ordered crystalline materials and amorphous disordered liquids over a wide temperature range. Moreover, PBLG belongs to the group of nematic mesophases, which show unidimensional order in the direction of the long rod-like shape molecular axes. This special characteristic allows LCPs, especially PBLG, to have a strong response to external influences such as an electrical field or magnetic field [29]. Lagerwall et al. [30] had proved that composite fibers with a core of nematic liquid crystal can be produced by coaxial ES while retaining aligned rod-like molecular structures. The research group suggested that core–shell fibers with an LCP as the core material can exhibit advanced functional characteristics. Following this idea, Cheng et al. [22,31] blended a ferroelectric polymer (PVDF) with a piezoelectric polymer (PMLG), and the electrospun PVDF/PMLG fibers exhibited significant enhancement of piezoelectricity. The voltage generated by the PVDF/PMLG fibers was nearly 2 times larger than that of pure PVDF or pure PMLG (185 mV compared to 105 mV and 80 mV, respectively). Therefore, in the experimental aspect, the combination of a piezoelectric polymer with a ferroelectric polymer has great potential as a functional material.

Electrospun core–shell nano/microfibers are an attractive trend in the study of nanogenerators and biosensors. D. Ponnamma et al. [32] fabricated core–shell nanofibers of PVDF-based nanocomposite which produced a good output voltage of 14 V. The PVDF-HFP/PVDF (core–shell) coaxial nanofibers containing ZnO and TiO_2_ nanoarchitectures generated a high output voltage at the excitation frequency of 45 Hz. Recently, Haowei Lu et al. [33] reported a remarkable high-performance piezoelectric nanogenerator produced from electrospun Tb-(BaCa(ZrTi)O_3_-PVDF coaxial fibers. The improved coaxial fiber with enhanced stress transfer generated an output of 48.5 V and 3.35 µA, values that are three times larger than those of conventional electrospun fibers. In the aspect of biosensors and implantable devices, the biocompatibility of biomaterials is an important characteristic. Narshiha Mamidi et al. [10,34] presented the application of nanofibers based on bovine serum albumin (BSA) for stimuli-responsive release of cargo in biomedical applications. However, in order to improve the electromechanical performance of biocompatible material, a new structure is still needed. In the present study, the influence of the ES process on the fabrication of coaxial fibers with PBLG as the core and PVDF as the shell material has been investigated. We report that there is the possibility of producing a new form of core–shell electrospun fibers composed of a biocompatible, large dipole moment based polymer and a ferroelectric polymer. The difference in the polarity direction of the core and shell material is capable of generating remarkable improvements in the electromechanical characteristics in both the axial and radial directions. The new type of core–shell fibers has promising functions for various types of applications such as sensors, actuators, smart materials, implantable biosensors, biomedical engineering devices, and energy harvesting devices.

This report is divided into five sections. The first section introduces the current state-of-the-art studies on piezoelectric fibers, especially the newly discovered piezoelectric polymer (PBLG) and the widely used ferroelectric polymer (PVDF). Section 2 describes the materials and fabrication methods in detail. Section 3 presents the characterization methods, including morphology, molecular orientation, and piezoelectric and electromechanical properties. More detailed results and discussion are presented in Section 4. Section 5 summarizes the results and suggests potential applications.

## 2. Materials and Methods

### 2.1. Materials

The polymer materials, poly(vinylidene fluoride) (average Mw~534,000, powder) and poly(γ-benzyl-α,L-glutamate) (Mw = 150,000–350,000, powder), were purchased from Sigma Aldrich (South Korea). N,N-Dimethylformamide (DMF, anhydrous, 99.8%) and dichloromethane (DCM, anhydrous, 99.8%) were used without any further purification steps. Acetone was mixed evenly with DMF at a volume ratio of acetone:DMF = 1:4 for 10 min using an ultrasonic stirring machine. Acetone was used to increase the evaporation rate during ES. PVDF was dissolved in a mixed solvent of acetone–DMF at a concentration of 30 wt%. The PVDF/DMF–acetone solution was kept at room temperature for 3 to 4 h to remove the microbubbles generated during the mixing step. The PBLG/DCM 10 wt% solution should be stored at 5 °C for 2–3 h before use. The storage period at low temperatures was observed to promote electrospinnability at the beginning of the jet.

### 2.2. Fabrication Method

**Figure 2:**
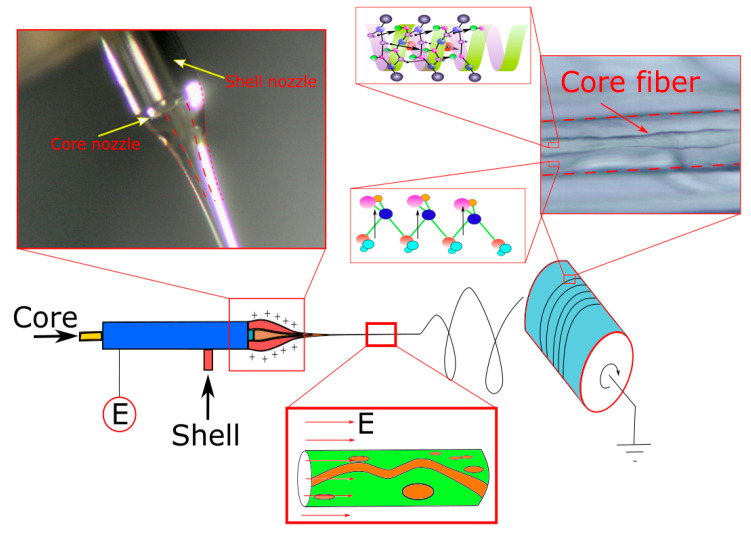
Schematic principle of fabrication of PBLG/PVDF core–shell fibers by electrospinning. The core solution is PBLG/DCM, the shell solution is PVDF/DMF–acetone. The external voltage (E) is from 16 to 20 kV.

**Figure 3:**
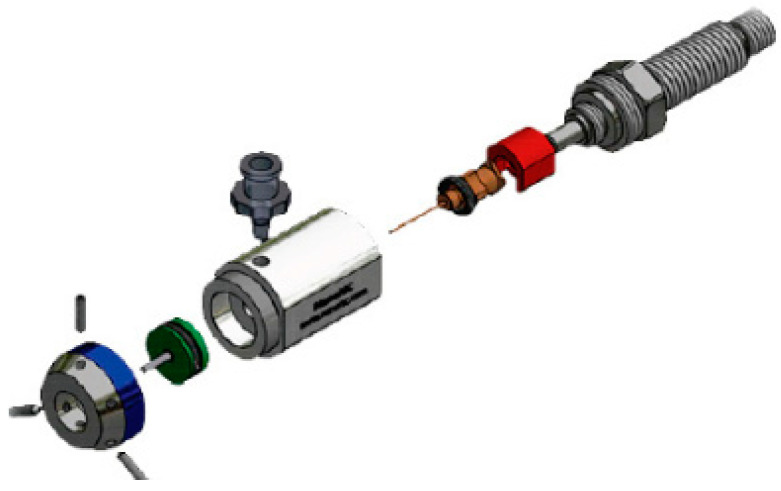
Exploded view of the coaxial nozzle, reprinted from NanoNC Ltd.

The fabrication setup consisted of two high-precision syringe pumps (New Era Pumping System NE-1000, New York, USA and Legato 110 Syringe Pump, KD Scientific, Massachusetts, USA), a high-voltage generator (Spellman SL60, USA), and a customized cylindrical collector (Figure 2). The coaxial nozzle was purchased from NanoNC (Seoul, Korea) and had an inner nozzle of G23 size and an outer nozzle of G17 size (Figure 3). The concentricity of the two nozzles could be adjusted precisely using a tri-point mechanism. The detailed conditions for the fabrication process are listed in Table 1. These conditions were established after several experimental trials. The fabrication process was observed to be the most robust after application of these conditions.

## 3. Results

### 3.1. Characterization Methods

#### 3.1.1. Morphology Characterization

**Figure 4:**
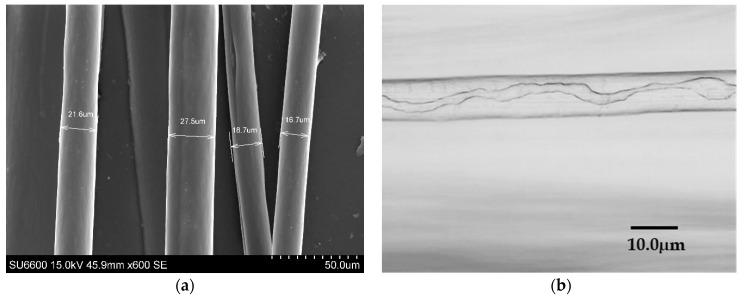
(**a**) The SEM micrograph of electrospun PBLG/PVDF fibers and (**b**) the core–shell structure.

The morphology of the electrospun fibers was characterized using scanning electron microscopy (SEM, SU6600 Field Emission; HITACHI, Japan). The SEM micrograph shows that the electrospun diameters varied from 15 to 30 μm (Figure 4a). An optical system was used to capture the core–shell structure (Figure 4b).

#### 3.1.2. Molecular Orientation Characterization

**Figure 5:**
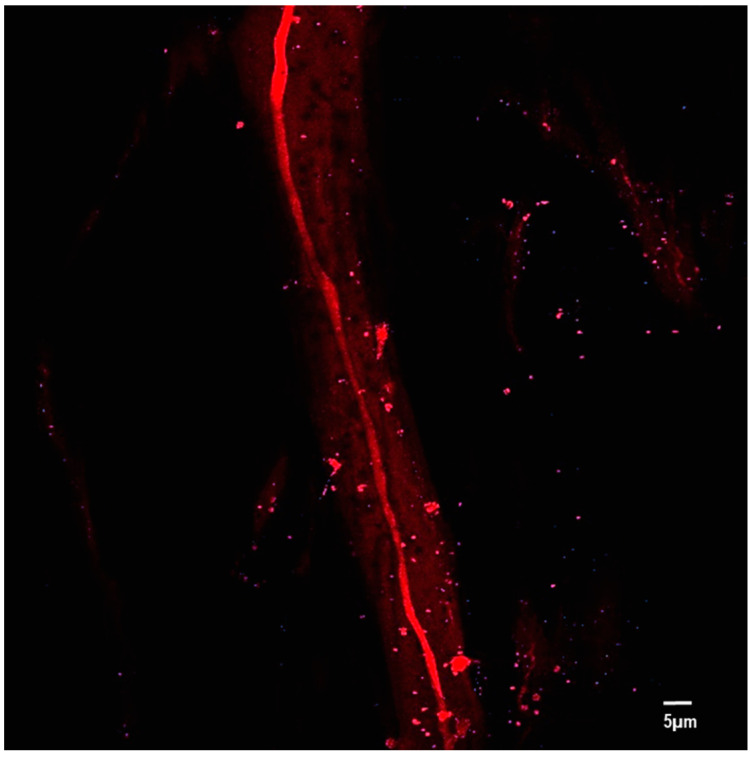
The SHG microscopy image of electrospun PBLG/PVDF fibers. The main red fiber is the core PBLG with a highly oriented dipole moment. The blurred red region is partially PVDF and/or a small amount of PBLG.

The second harmonic generation (SHG) method has been used to check the polarity of nonlinear optical materials such as PBLG [25,28]. The SHG method is an effective way to determine the polarization of polymers consisting of large and parallel chain molecular dipole moments. The existence of both parallel and antiparallel dipole moments within a single PBLG molecule is possible, which could greatly reduce the overall piezoelectricity [35]. It has been proven that strong and stable parallel dipole moments inside PBLG molecules are formed under a high electric field. Block et al. [36] revealed that a PBLG film poled by an external field retained the aligned molecular dipole moment for over 6 months. Farrar et al. [25] and Nguyen et al. [28] validated that the dipole moment in electrospun PBLG fibers is polarized in a parallel orientation. The same analysis method was employed to study the electrospun coaxial PBLG/PVDF fiber bundle using an SHG microscopy system (LEICA TCP SP5) at a wavelength of 900 nm and a power source of 2454 W (Figure 5).

#### 3.1.3. Piezoelectric Characterization

Piezoelectric coefficients can be measured based on either direct or indirect effects. The direct effect of a piezoelectric material corresponds to an electrical signal (accumulated charges or difference in voltage at the electrodes) generated by a load or mechanical pressure along the orientation of the dipole moment. The indirect effect is the reverse phenomenon of the direct effect; however, it is usually more difficult to achieve precise measurements by an indirect effect than a direct effect. Because of the notation difference between the piezoelectric mode of PBLG and PVDF fibers, the piezoelectric direction notation of the coaxial fibers follows that of the PBLG fiber, that is,

d_33_coaxial_ = d_33_PBLG_ = d_31_PVDF_

d_13_coaxial_ = d_13_PBLG_ = d_33_PVDF_

**Figure 6:**
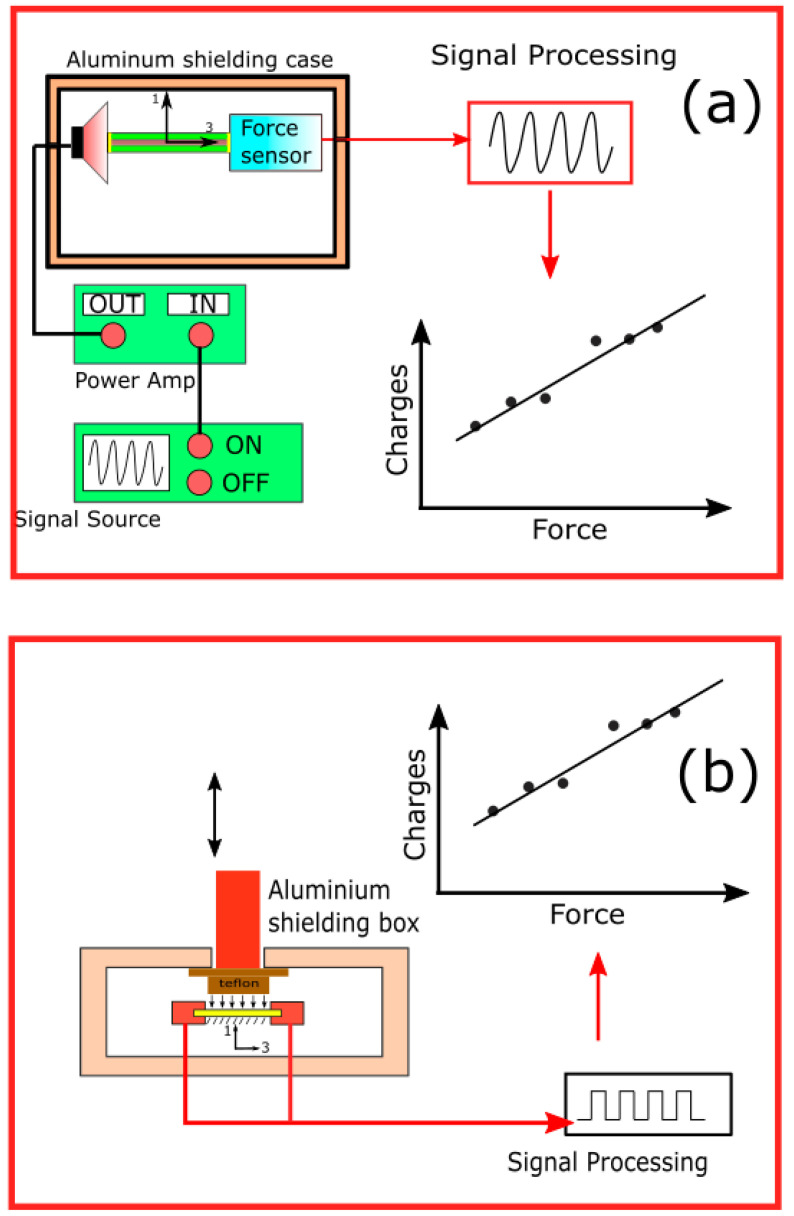
Experimental setup to measure the piezoelectric coefficients (**a**) d_33_ and (**b**) d_13_.

In this study, a measurement setup similar to that reported previously in the literature has been used [26]. Each sample was 30 mm long, 5 mm wide, and approximately 200 µm thick. The width of the bundle was manipulated using the end-point control assembly (EpCA) method [37]. A total of 50 samples were used in the experiments. The electrodes were coated with a 150 nm thick Pt layer via sputtering. The piezoelectric 33-mode means that the mechanical extension load and electrode are applied along the longitudinal direction (Figure 6a). The core–shell fibers were clamped at the two ends along the axial directions using a loudspeaker baffle and a high-sensitivity force sensor (FUTEK LSB2000; Irvine, CA, USA). The loudspeaker acted as the actuator and was controlled by a signal source. The loudspeaker baffle allowed elongation of the sample at high frequency, small stroke, and easy setup. The overall system was shielded by a grounded aluminum box to prevent noise. By adjusting the amplitude and frequency, the different levels of force applied to the sample could be measured. The elongation force and the measured charges were recorded simultaneously using the NI-USB-6361 X-Series Multifunction DAQ and processed using a customized LabVIEW program (National Instruments, Texas, USA). The d_33_ value was calculated as the ratio of charge over a constant level of extension force. The d_13_ value was measured by applying the load in the transverse direction (1-direction) and measuring the charges between the two ends of the fibers in the longitudinal mode (3-direction) (Figure 6b). The load was calibrated using standard weights (100, 200, and 500 g). To prevent any noise in the charge measurement, all devices were placed in an aluminum shielding box, and the standard weight imposed pressure on the sample through a Teflon-based film.

#### 3.1.4. Electromechanical Response

To test potential applications such as energy harvesting devices or force sensors, the electromechanical response was recorded in a configuration similar to that of the piezoelectric coefficient measurement (Figure 6). Instead of measuring the charge outcome, the electromechanical response involved the measurement of the generated output voltage under the application of a certain level of load. The output voltage along the electrospun PVDF and PBLG fiber axes was observed to be significantly improved [20,21,26]. Therefore, the electromechanical response was recorded mainly in the longitudinal direction. The electromechanical response was measured under actuation frequencies of 20, 40, and 60 Hz, which were equivalent to the measurements with electrospun PBLG fibers reported by Cheng et al. [31] and Nguyen et al. [26].

## 4. Discussion

### 4.1. Morphology and the Fabrication Condition of Core–Shell PVDF/PBLG Fibers

Figure 4a shows that the shell fiber diameter varied from 15 to 30 µm while the core fiber diameter varied from 1 to 5 µm. The core–shell structure in Figure 4b reveals several limitations of the current fabrication configuration regarding the twisted form of the core. Because of the surface tension of the PVDF solution, the core PBLG solution was unable to gradually elongate along the axial direction to produce a sub-micrometer thin, uniform, and straight inner core. Instead, it twisted inside the shell PVDF jet during elongation. This can be explained by the strong reaction of the PBLG molecules under the electric field, which causes the PBLG jet to eject from the shell jet. Meanwhile, the viscosity and surface tension of the shell solution prevented this ejection phenomenon. The thick fiber diameter can be explained by the fabrication parameters such as the relatively close distance of the collector to the nozzle (less than 7 cm), the high viscosity caused by the high concentration of the shell solution (PVDF/DMF–acetone), and the size of the outer nozzle (G17 size, outer diameter (OD) = 1.47 mm) being 2 times larger than that of the conventional single-spinneret ES (from G25 to G30, OD = 0.3 mm). The other important factor was the interaction of the inner fluid (PBLG/DCM) with the external field. In the single-nozzle ES, the thinning or elongation process of a solution is caused by the evaporation of the solvent, incorporating the shear force in the radial direction given by the Coulomb force. However, in the coaxial fabrication process, the PBLG jet is confined by a PVDF solution with extremely high viscosity and surface tension, and DCM is not exposed to the environment, which causes a low evaporation rate of the inner jet. Therefore, to shrink the fiber diameters to nanoscale or sub-microscale, more work needs to be done in the future to determine the optimal fabrication conditions.

### 4.2. Molecular Orientation

Figure 5 shows that the core PBLG exhibits an intense red color, which originates from the nonlinear optical properties of the chiral liquid crystal polymer when the molecule is oriented along the axial direction. In contrast to the saturation signal produced by the core PBLG, the shell PVDF only reflects blurred regions, which are due to the lower molecular crystal orientation levels in the axial direction. In other words, the SHG experiment revealed that electrospun PVDF fibers contained a minor number of molecularly oriented fibers in the fiber axial directions, which was also verified by Xia Liu et al. [38] and T. Lei et al. [12]. The red spots in Figure 5 may be due to partial de-emulsification phenomena caused by the elongation process, the “inner jet erection” phenomena during the whipping process caused by the relatively stronger interaction of the dipole PBLG molecule, and the nonuniform diffusion of the PBLG/DCM solution in the PVDF/DMF–acetone solution. Therefore, at the collector, a portion of PBLG beads with sub-micrometer diameters was collected along with coaxial PBLG/PVDF fibers with diameters of several tens of micrometers (Figure S1).

### 4.3. Piezoelectric and Electromechanical Response Measurement

**Figure 7:**
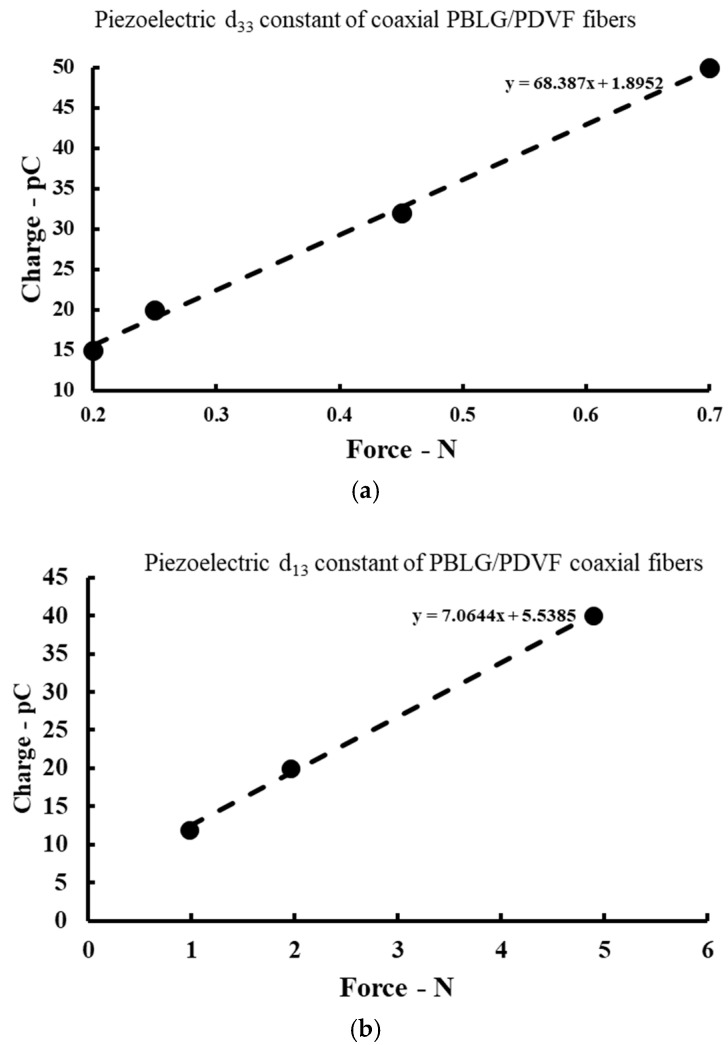
(**a**) The graph of the d_33_ coefficient with the fitting line. The R^2^ value is 0.9533. (**b**) The graph of the d_13_ coefficient with the fitting line. The R^2^ value is 0.9612.

The average piezoelectric coefficient d_33_ of coaxial fibers was found to be 68.3 pC N^−1^, which is much higher than that of pure homopolymer electrospun PBLG and PVDF fibers (Table 2), while the piezoelectric coefficient d_13_ was determined to be 7 pC N^−1^ (Figure 7). The improvement in the d_33_ value may result from the combination of the polarity effect of PVDF and PBLG, while the small d_13_ value was probably caused by the nonuniform contribution of fibers in the bundle. In other words, when the transverse load was presented, it was not distributed evenly across all fibers, which led to the nonuniform deformation of the fibers. This is the first time that a coaxial fiber composed of two different piezoelectric polymers with different polarity characteristics has been fabricated and characterized. Cheng-Tang Pan et al. [39] applied near-field electrospinning to fabricate ordered PVDF/PMLG composite fibers to produce a flexible energy harvester with a peak voltage of 0.08 V and a power of 637.81 pW [40]. The research group verified that the fraction of α-helical conformation of PMLG increased continuously with increasing PVDF content. The authors suggested a scheme for conformational changes and interactions between PVDF and PMLG fibers (Figure S2). After polarization in a high electric field, the wide-angle X-ray diffraction (WAXD) and Fourier transform infrared (FTIR) spectroscopic analyses showed that the α-helical structure of the PMLG molecules was stabilized, while the intramolecular hydrogen bond interactions between PLMG and the PVDF chain increased significantly. It is noteworthy that PBLG and PMLG have similar conformation structures. Therefore, the enhancement of the piezoelectric coefficient of PVDF/PBLG may have similar fundamentals as that of PVDF/PMLG. However, more comprehensive studies should be conducted to investigate the detailed interactions in the multiphase region and even at the molecular level.

**Figure 8:**
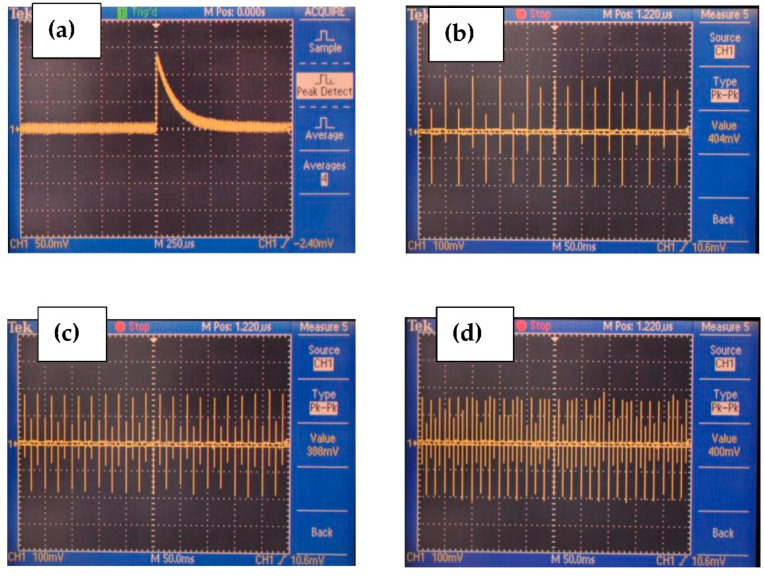
The voltage measured under bending force to the coaxial PBLG/PVDF fibers at different frequencies: (**a**) pulse signal at 20 Hz, (**b**) 20 Hz, (**c**) 40 Hz, and (**d**) 60 Hz.

**Figure 9:**
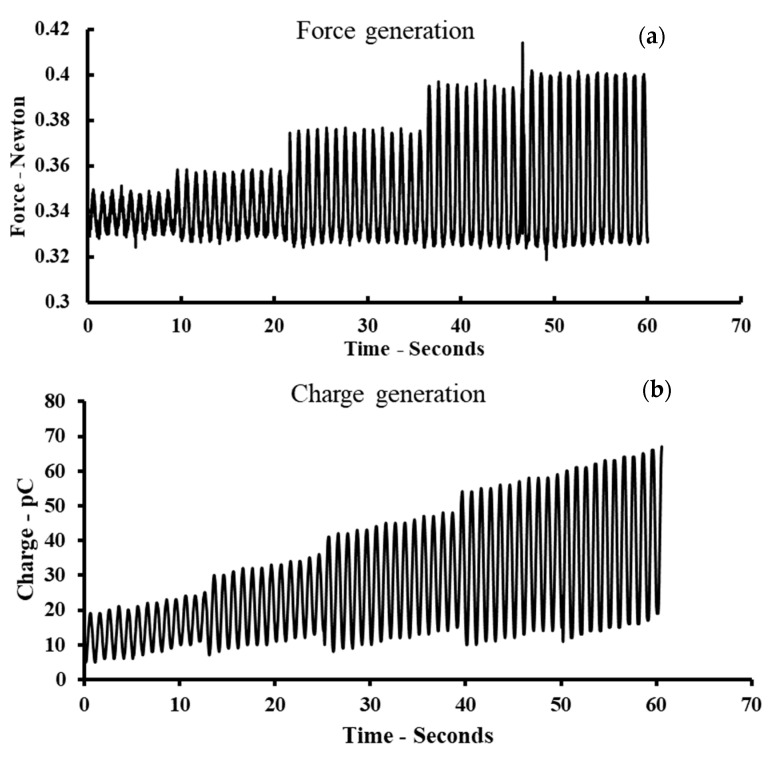
The force applied (**a**) by a sine shape form along the fiber axes and (**b**) charge generation in time domain.

The generated voltage measured by an oscilloscope showed a consistent signal up to 400 mV peak-to-peak, which is significantly higher than that of homopolymer fibers or composites such as PBLG/PDMS or PVDF/PDMS (Figure 8). The pulse signal was generated at 20, 40, and 60 Hz in the longitudinal direction. The results suggest the good reliability of the proposed composite for measuring the dynamic pressure force in various applications such as pressure sensors and energy harvesting devices. The detailed setup and results are presented in the supporting video (Video S1). Figure 9 shows the charge generation corresponding to the force applied by a sinusoidal function along the fiber axes. The slope trend in Figure 9b has been inherited from the charge-drifting phenomenon, which is unavoidable in high-precision measurements such as electrostatic charge measurements. The equivalent trend of charge generation with respect to the applied force suggests the potential applications of the PBLG/PVDF composite in electromechanical sensors or force sensors at high frequencies.

## 5. Conclusions

The study presents achievements in the field of flexible piezoelectric polymer fabricated by the electrospinning method. The high electrostatic field and high mechanical elongation rate promote the formation of the dipole moment and increase the polar phase in both PBLG and PVDF structures. The integration of two different piezoelectric and ferroelectric polymers to produce core–shell microfibers has been successfully implemented. By the use of the electrospinning fabrication method, an oriented core–shell structure with a fiber diameter of 15–30 µm was fabricated. The SHG method was used to analyze the orientation of the core PBLG fibers, verifying the hypothesis that most of the dipole moments were parallel. The highly oriented dipole moment of PBLG guarantees a significant improvement in electromechanical performance. The piezoelectric coefficient measurements were conducted and showed significant enhancement of the PBLG/PVDF core–shell fibers up to 68 pC N^−1^ in the 33-mode and 7 pC N^−1^ in the 13-mode. Compared to conventional electrospun fibers, the piezoelectric coefficient of the axial axis has been increased 2.5 times, showing a great potential to develop flexible electromechanical transducers. In addition, the electromechanical measurements depicted a stable output voltage of 400 mVpp in the frequency range of 20 Hz to 60 Hz. Further experiments need to be conducted to elucidate the characteristics of the core–shell structure, such as differential scanning calorimetry (DSC) analyses to confirm the miscibility at the molecular level and FTIR spectroscopy to analyze the chemical composition and the chemical reaction in the multiphase region. The enhanced electromechanical properties of coaxial electrospun core–shell PBLG/PVDF fibers suggest their potential applications in sensors, actuators, smart materials, implantable biosensors, biomedical engineering devices, and energy harvesting devices.

## Figures and Tables

**Table 1:** The coaxial PBLG/PVDF microfiber fabrication procedure.

Parameter	Unit	Value
PBLG/DCM concentration	wt%	10
PVDF/DMF–acetone (4:1) concentration	wt%	30
Feeding rate of core solution	μL/min	0.15
Feeding rate of shell solution	μL/min	0.05
Tip-to-collector distance	cm	5–7
Collector rotation speed	rpm	3000
Voltage	kV	16–20

**Table 2:** Properties of several piezoelectric polymers and fiber-based composites [9].

Material	Piezoelectricity (pC N^−1^)	Maximum Voltage Output (mV)
PVDF	d_31_ = 8–22 d_33_ = −24 to −34	85
P(VDF-TrFE)	d_31_ = 12	1500
Poly-L-lactide	d_14_ = −1.83	-
Liquid crystal elastomers	d_33_ = −70	-
PBLG	d_33_ = 27 d_31_ = 0.1	65
PVDF/PDMS composite	d_33_ = 34 d_31_ = 19	190
PBLG/PDMS composite	d_33_ = 54 d_31_ = 10.2	200
PBLG/PVDF coaxial fibers	d_33_ = 68 d_31_ = 7	400

## Data Availability

The data presented in this study are available on request from the corresponding author.

## Supplementary Materials

The following supporting information can be downloaded at: https://www.mdpi.com/article/10.3390/polym14091739/s1, Figure S1: SEM photograph of electrospun bundles fibers with core-shell fibers, single PBLG fibers and the beads; Figure S2: Conformation changes and specific interaction between PVDF and PMLG fibers—suggested by Cheng-Tang Pan et al.; Video S1: Video Supplementary_Force vs voltage generation_demonstration.
