# Supplementary material for: Significant Electromechanical Characteristic Enhancement of Coaxial Electrospinning Core–Shell Fibers
Source: Polymers (Basel). 2022 Apr 25;14(9):1739. doi: 10.3390/polym14091739 (PMC9099492; doi:10.3390/polym14091739)
Supplement: Supplementary file 1 [file polymers-14-01739-s001.zip › SUPPORTING INFORMATION.pdf]

SUPPORTING INFORMATION

Research Article

Significant electromechanical characteristic enhancement of coaxial electrospinning core-shell fibers

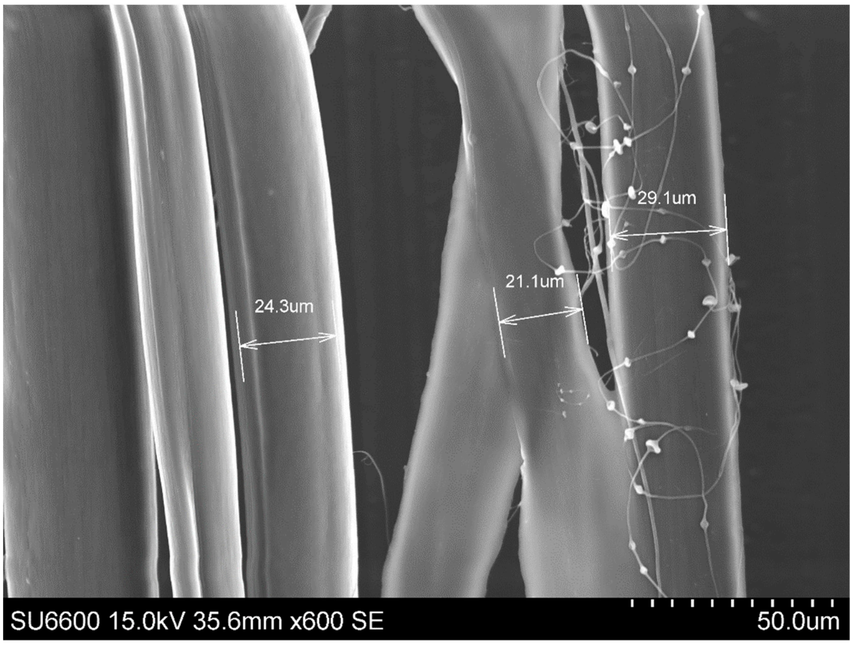

Figure S1. SEM photograph of electrospun bundles fibers with core-shell fibers, single PBLG fibers and the beads.

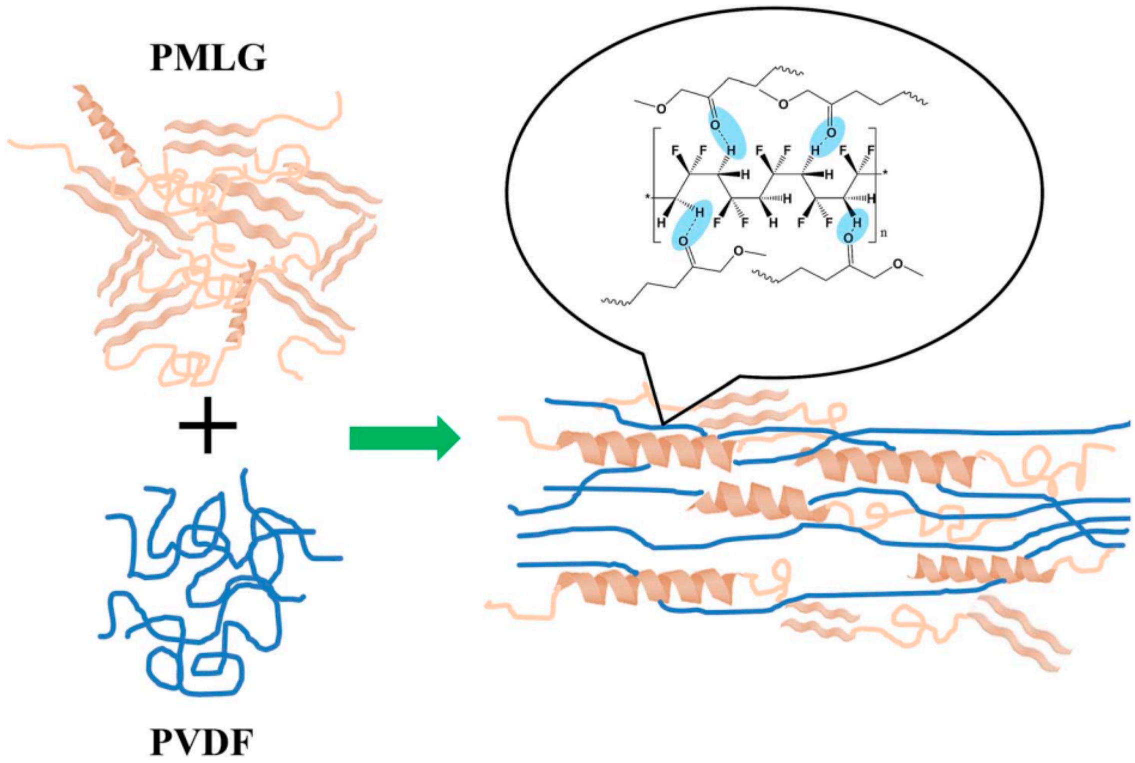

Figure S2. Conformation changes and specific interaction between PVDF and PMLG fibers – suggested by Cheng-Tang Pan *et al*
